# Contribution of “Omic” Studies to the Understanding of Cadasil. A Systematic Review

**DOI:** 10.3390/ijms22147357

**Published:** 2021-07-08

**Authors:** Elena Muiño, Israel Fernández-Cadenas, Adrià Arboix

**Affiliations:** 1 Stroke Pharmacogenomics and Genetics Group, Institut de Recerca de l’Hospital de la Santa Creu i Sant Pau, 08041 Barcelona, Spain; elena.muinho@gmail.com; 2 Cerebrovascular Division, Department of Neurology, Hospital Universitari del Sagrat Cor, Universitat de Barcelona, 08007 Barcelona, Spain

**Keywords:** CADASIL, genomic, transcriptomic, proteomic

## Abstract

CADASIL (Cerebral Autosomal Dominant Arteriopathy with Subcortical Infarcts and Leukoencephalopathy) is a small vessel disease caused by mutations in *NOTCH3* that lead to an odd number of cysteines in the epidermal growth factor (EGF)-like repeat domain, causing protein misfolding and aggregation. The main symptoms are migraines, psychiatric disorders, recurrent strokes, and dementia. Omic technologies allow the massive study of different molecules for understanding diseases in a non-biased manner or even for discovering targets and their possible treatments. We analyzed the progress in understanding CADASIL that has been made possible by omics sciences. For this purpose, we included studies that focused on CADASIL and used omics techniques, searching bibliographic resources, such as PubMed. We excluded studies with other phenotypes, such as migraine or leukodystrophies. A total of 18 articles were reviewed. Due to the high prevalence of *NOTCH3* mutations considered pathogenic to date in genomic repositories, one can ask whether all of them produce CADASIL, different degrees of the disease, or whether they are just a risk factor for small vessel disease. Besides, proteomics and transcriptomics studies found that the molecules that are significantly altered in CADASIL are mainly related to cell adhesion, the cytoskeleton or extracellular matrix components, misfolding control, autophagia, angiogenesis, or the transforming growth factor β (TGFβ) signaling pathway. The omics studies performed on CADASIL have been useful for understanding the biological mechanisms and could be key factors for finding potential drug targets.

## 1. Introduction

CADASIL (Cerebral Autosomal Dominant Arteriopathy with Subcortical Infarcts and Leukoencephalopathy; OMIM#125310) is a systemic arteriopathy of non-atherosclerotic and non-amyloid cause. It is a rare disease affecting fewer than 2/1000 individuals, caused by mutations in the *NOTCH3* gene. It has autosomal dominant inheritance, although it can also occur due to *de novo* mutations [1]. 

The etiopathogenesis of the disease is not well understood. It is thought to be triggered by mutations in *NOTCH3* that cause an odd number of cysteines in the domain hosting the epidermal growth factor-like repetitions (EGFr) of the receptor encoded by this gene, leading to disruption of disulfide bonds and protein aggregation [2,3].

CADASIL is characterized by the following symptoms: migraine with aura; psychiatric disorders; recurrent small subcortical infarcts; and dementia at an early age [4]. It is the most common cause of stroke and dementia of genetic origin. On MRI scans, white matter hyperintensities (WMH) in the temporal lobe and external capsule are characteristic of the disease [5,6].

The presence of protein aggregates known as granular osmiophilic material (GOMs) in skin biopsies of patients, assessed by electron microscopy, has 100% specificity for its diagnosis [5]. However, due to the focal nature of GOMs, false negatives may occur [7]. Therefore, the definitive diagnosis is established through genetic testing with the identification of pathogenic mutations, which affect the number of cysteines in EGFr.

Once the diagnosis has been made, it is difficult to determine the patients’ clinical course. Patients can progress differently, even if they have the same mutation, belong to the same family, or even if they are monozygotic twins [8,9].

There is no curative or disease-modifying treatment, only symptomatic treatments are available. Hence the importance of deepening our understanding of the disease in order to find therapeutic targets whose modulation can improve the quality of life of these patients.

Omic technologies are used to detect genes (genomics), mRNA (transcriptomics), proteins (proteomics), and metabolites (metabolomics) in a specific biological sample in a non-targeted and non-biased manner. The integration of these techniques is called systems biology [10]. Unlike with traditional studies, with these techniques, it is possible to generate hypotheses, which are mostly driven or reductionist hypotheses [10]. 

Omic technologies can be used in screening, diagnosis, and prognosis, as well as for aiding our understanding of the etiology of diseases or identifying biomarkers [10,11]. Moreover, they are used in target [12] and drug discovery and in the assessment of their toxicity and efficacy [10]. 

With this review, we aim to take a closer look at the progress made in recent years in CADASIL thanks to omics technologies, as well as to explore what therapeutic possibilities these technologies could offer through a comprehensive resource for omics research on drugs, such as the DrugBank (https://go.drugbank.com, accessed on 27 May 2021). 

The use of omics technologies in the field of CADASIL allows for a more efficient diagnosis of the disease. From an epidemiological point of view, it has been found that in genomic repositories, there is a high prevalence of individuals with variants affecting the number of EGFr cysteines. This then raises the question of whether they all really are pathogenic mutations, which was previously considered to be the case. From an etiological point of view, the massive study of data has highlighted the importance of metabolic functions/pathways related to the extracellular matrix, cell adhesion, autophagy, misfolding control, angiogenesis, or TGFβ signaling. These technologies have allowed us to understand the histopathological findings in the disease or identify which molecules or pathways may be of interest for drug targeting, opening a wide range of possibilities for the development of future clinical trials.

## 2. Materials and Methods

We have conducted an exhaustive literature search up to April 2020 by one reviewer in PubMed, LILACS, Trip Database, and The Cochrane Library. The keywords used were: (CADASIL[Title/Abstract]) AND ((proteom*[Title/Abstract]) OR (tran-scriptom*[Title/Abstract]) OR (genom*[Title/Abstract]) OR (gwas[Title/Abstract]) OR (integrom*[Title/Abstract]) OR (metabolom*[Title/Abstract]) OR (microbio*[Title/Abstract])).

A total of 58 articles was listed in PubMed, zero in LILACS, 23 in Trip Database and one in The Cochrane Library. Fifty-six articles were excluded as they were not relevant to our study.

**Figure 1:**
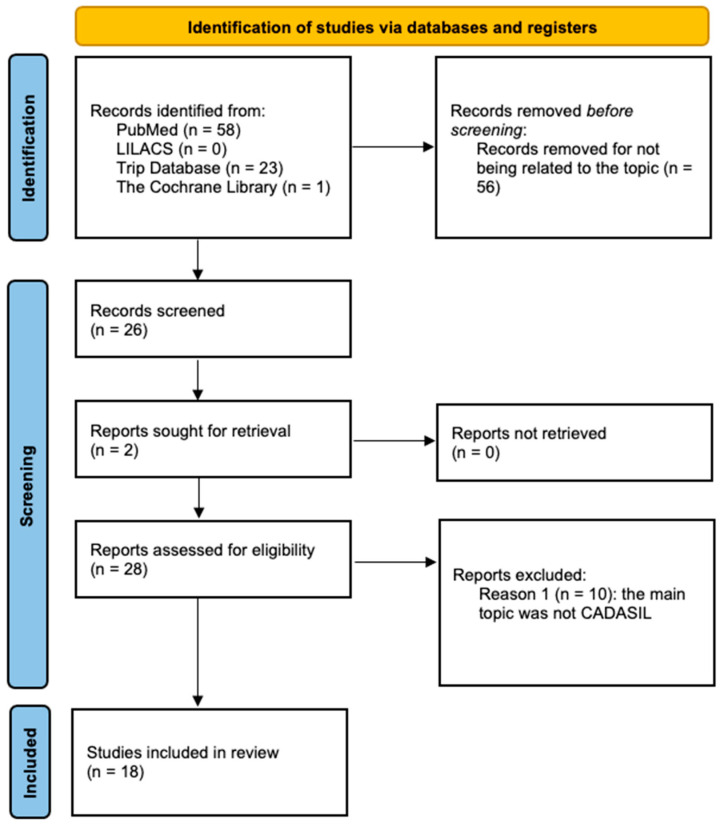
PRISMA flow diagram.

Twenty-six articles were screened and two more were sought for retrieval. Ten articles were excluded because the topic was not on CADASIL, and they focused on other phenotypes, such as migraine or leukodystrophies. Finally, we reviewed 18 studies (Figure 1).

For the search for the GO molecular function and biological process we used *Ensembl*. We use DrugBank (https://go.drugbank.com) to discover which potential drugs could interfere with the molecules obtained with omics technologies. DrugBank is a comprehensive resource for omics research on drugs. It offers drug-action pathways, drug transporter data, drug metabolite data, pharmacogenomic data, adverse drug response data, ADMET data, pharmacokinetic data, computed property data and chemical classification data.

## 3. Genome-Wide Sequencing and Progress in Epidemiology

The first efforts for detecting the true prevalence of CADASIL were made in 2005, with an attempt at estimating the minimum prevalence of this disease in the East of Scotland [13]. By studying exons 3, 4, 5, and 6 of the *NOTCH3* gene or skin biopsies of patients with suspected CADASIL, the minimum prevalence was established at 1.98/100,000 adult individuals. In 2012, a similar low prevalence was confirmed in the North East of England (1.32/100,000 adults) [14]. Later studies found a minimum prevalence in Scotland and Central Italy that was almost twice as high: 4.1/100,000 adult individuals [15,16].

With the improvement of technology that allows for the massive study of data, diagnosis of the disease has become more efficient. It is now possible to analyze the entire *NOTCH3* gene in less time and at a lower cost, instead of studying only certain exons or even clinical exomes.

**Figure 2:**
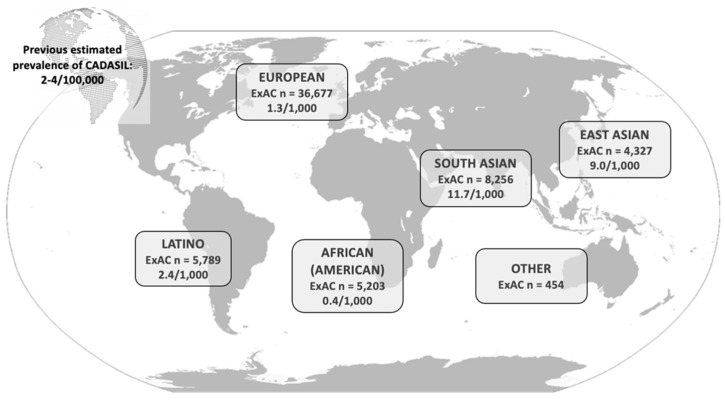
Graphical representation of the frequency of pathogenic CADASIL mutations according to the populations included in the ExAC database (currently called gnomAD). Figure adapted from Rutten et al. (2016) [17].

In fact, because of the creation of biobanks, which are projects that aggregate and harmonize exome and genome sequencing data from a wide variety of large-scale sequencing studies, it has been possible to further profile mutations affecting cysteine residues in Notch3 in a significant number of individuals. In the Genome Aggregation Database (gnomAD, http://gnomad.broadinstitute.org, accessed on 27 May 2021), the UK Biobank (UKB, https://www.ukbiobank.ac.uk, accessed on 27 May 2021) and the Geisinger DiscovEHR (http://www.discovehrshare.com, accessed on 27 May 2021), a prevalence of 1.4–3.4/1000 subjects carrying *NOTCH3* variants that were considered pathogenic were established [17,18,19,20,21] (Figure 2), and 9/1000 in the Taiwan Biobank (https://taiwanview.twbiobank.org.tw/index, accessed on 27 May 2021) [22], the latter high frequency is in line with the UKB observation of enrichment of pathogenic mutations in *NOTCH3* in Asians [19].

**Figure 3:**
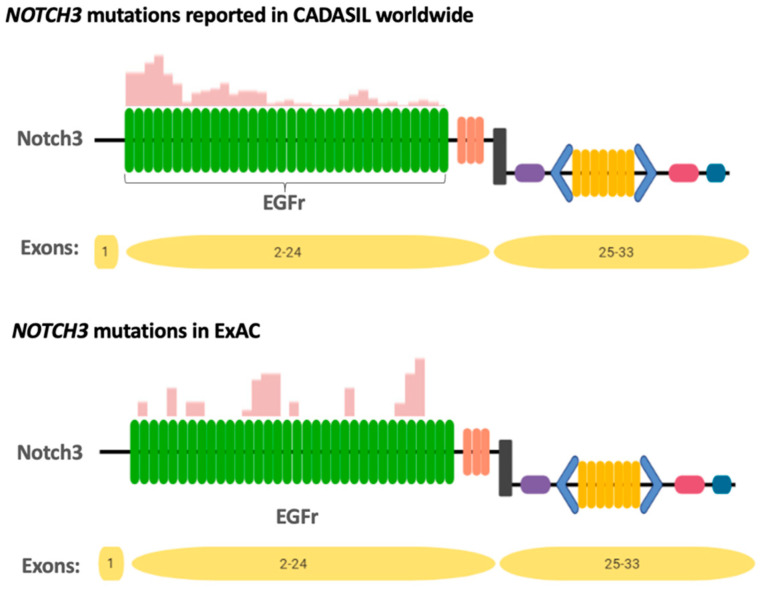
Distribution of different EGFr cysteine altering *NOTCH3* mutations in ExAC (currently called gnomAD) compared with those reported in cerebral autosomal dominant arteriopathy with subcortical infarcts and leukoencephalopathy (CADASIL) patients. Figure adapted from Rutten et al. (2016) [17].

This progress led some authors to hypothesize that there may be paucisymptomatic phenotypes due to the different location of the mutations within the protein, as the profile of EGFr affected in patients diagnosed with CADASIL and population cohorts is different (Figure 3) [17,18,19].

## 4. Omic Studies and Progress in the Etiopathogenesis of CADASIL

### 4.1. Proteomic Studies and Progress in the Etiopathogenesis of CADASIL

Multiple efforts have been made to try to understand the etiopathogenesis of this disease. The most widely accepted hypothesis was that the presence of an odd number of cysteines in the EGFr region leads to disruption of disulfide bonds and aggregation of the extracellular receptor domain (ECD) of both the Notch3 produced by the mutant allele and the Notch3 produced by the wild-type (WT) allele, as well as other proteins [2,23]. The formation of these complexes can be intracellular, at the plasma membrane of vascular smooth muscle cells (VSMCs) and pericytes [24,25], and extracellular in the so-called GOMs [26,27].

In the pre-omics era, most studies focused on the histological analysis and functional study of the receptor. It became clear that cellular adhesion to the extracellular matrix and between cells is disrupted, with degeneration and loss of endothelial cells, VSMCs, and pericytes [28,29,30,31,32,33,34,35], leading to altered autoregulation of blood flow and cerebral perfusion [31]. Besides, it has been recently postulated that there is an increase in Notch3 activity [36,37], hypothesizing that ECD aggregation could lead to aberrant Notch3 activation (independent of ligand binding).

On the other hand, only a few studies have explored other possible alterations that could influence the development of the disease. Some studies revealed that autophagy could be affected, as Notch3 accumulation is a possible indicator of autophagolysosomal pathway deficiency, as well as impaired intracellular trafficking and lysosomal maturation or defects [38]. Other studies showed the possibility of mitochondrial changes, as histological studies revealed mitochondrial morphological changes in VSMCs [33,39,40,41], or sporadic cases have shown altered mitochondrial respiratory chain complexes [42]. 

However, because of the omics sciences and genetic data repositories, it has become clear that there is a high prevalence of these types of mutation that are considered pathogenic, which may suggest that some are a risk factor for small vessel disease and that not all of them actually produce CADASIL [19,20,21,22]. On the other hand, if all of them do cause CADASIL, it should no longer be considered a rare disease.

In fact, the etiopathogenesis of the disease has been elucidated in more detail. Arboleda-Velasquez et al. analyzed the tunica media of cerebral arteries from two CADASIL patients and two sex- and age-matched controls post-mortem [43]. Using laser capture microdissection and mass spectrometry (MS), they found 19 proteins differentially expressed in CADASIL vs. control samples (Table 1). When we searched Gene Ontology (GO), it became apparent that these 19 proteins were involved in cell adhesion processes, cytoskeleton or extracellular matrix components, misfolding control, and clearance. This finding is in line with histopathological alterations found in pathology studies and highlights other pathways of interest, such as autophagy.

Monet-Leprêtre et al. used nano-liquid chromatography-tandem MS to study human brain artery samples from one CADASIL patient and one healthy donor. They found 104 enriched proteins in the CADASIL sample, the majority of which was in the extracellular matrix proteins. The Notch3 ECD was almost undetectable in the control but vastly accumulated in the CADASIL sample [44]. Other molecules related to matrix metalloproteinases were found in proteomic experiments with animal models. Murine brain arteries of TghNotch3 (WT) and mutant transgenic TghNotch3 (R90C) mice were analyzed. Two proteins, TIMP3 (belonging to the TIMP family, which are metalloproteinase inhibitors) and vitronectin (VTN, a cell adhesion regulator, signaling cytoskeleton reorganization and extracellular matrix homeostasis [44,45]), were present in the mutant arteries and almost absent in the control arteries. Moreover, these proteins were strongly enriched in the human CADASIL sample experiment and were recruited into CADASIL deposits in vivo [44]. 

On the other hand, of the 19 proteins identified by Arboleda-Velasquez et al., 11 were analyzed by Monet-Leprêtre et al., supporting the previous results.

Zellner et al. performed a brain vessel proteome with MS with six CADASIL patients (carrying five different *NOTCH3* mutations) and six age-matched neurologically healthy controls. Of the quantified proteins, 190 showed a raw *p*-value <0.05 [46]. Categorization of proteins according to subcellular localization (UniProt database) demonstrated a strong overrepresentation of extracellular space, secreted, and mitochondrial proteins. Most of the mitochondrial categories were depleted in the CADASIL group, and the majority of secreted and extracellular space proteins were enriched [46]. Notch3 exhibited the strongest increase in abundance, and multivariate analysis showed significance for Notch3 and 16 additional proteins with increased abundance [46] (Table 1).

Again, most of the molecular functions and biological processes (Gene Ontology) of these proteins are involved in cell adhesion processes, extracellular matrix components, angiogenesis, and the TGFβ signaling pathway.

As the HTRA1 protein was the most enriched protein, in this study, the authors analyzed its role in CADASIL. First, they proved that there was no mutation in *HTRA1* DNA of CADASIL patients. Additionally, they found that the abundance of the protein did not correspond to differences between cases and controls in their mRNA, and that HTRA1 was located close to Notch3 deposits [46]. They also discovered that several of the proteins found in high numbers in CADASIL patients are substrates of HTRA1 (cell migration-inducing and hyaluronan-binding, olfactomedin-like 3, semaphorin3G, and chordin), in addition to the already known substrates, such as VTN, an observation compatible with a reduction in its proteolytic activity [46].

Change AV (article of Arboleda-Velasquez et al. [43]), ML (article of Monet-Leprêtre et al. [44]), N (article of Nagatoshi et al. [47]): + in the case of a higher protein level in the samples of patients with CADASIL; − in the case of a lower protein level in samples from CADASIL patients compared with controls. Molecular functions and biological process: some of the molecular functions and biological processes from Gene Ontology for each protein. For a better understanding, we have grouped related terms together. Nagatoshi et al. used laser microdissection to collect GOM-enriched leptomeningeal arteries from two autopsied CADASIL patients and superficial temporal arteries from one biopsied CADASIL patient, and five controls [47]. Liquid chromatography–tandem MS showed that Notch3, serum amyloid P-component, annexin A2, and periostin, exhibited the largest increase [47]. Once again, our research on GO terms showed that most of the proteins detected by Nagatoshi et al. were involved in cell adhesion processes, cytoskeleton or extracellular matrix components, angiogenesis, or clearance. 

Proteins from this study that were found in the article by Zellner et al. all followed the same direction, except for gelsolin and plectin. The histopathological analysis showed co-localization of serum amyloid P component and Notch3, predominantly in the tunica media of the vessels. ELISA analysis showed that serum amyloid P component biochemically binds to Notch3 dose-dependently, but not to Notch1 or Notch2 [47]. Interestingly, the expression study by RT-PCR with primers detecting serum amyloid P component in the liver showed low expression in both CADASIL patient samples and controls, suggesting that perhaps the marked reaction of its antibody in the cerebral vascular wall was due to cerebral synthesis. On the other hand, the authors found no difference between blood protein levels of serum amyloid P component between CADASIL patients and controls. The two other proteomic studies mentioned above [43,44] showed the same direction of effect of serum amyloid P component, strengthening this result.

Primo et al. generated a proteome using an aorta and blood taken from CADASIL mice with 53 angiogenesis-related proteins, pointing out that col18α1/endostatin could be a potential biomarker [48]. In addition, these authors explored the mice plasma levels of HTRA1 and Notch3 ECD as candidate biomarkers, finding an increase in the former and a decrease in the latter [48]. 

These studies discovered the presence of several proteins located next to *NOTCH3*, and this has allowed the in-depth study of the composition of GOMs, which are a hallmark of the disease.

### 4.2. Transcriptomic Studies and Progress in the Etiopathogenesis of CADASIL

A transcriptomic and proteomic study was performed on post-mortem brain material (frontal cortex and white matter) from two unrelated cases of CADASIL and five controls. With regard to changes in gene and protein expression, the authors found many terms related to RNA metabolism, such as RNA processing; RNA degradation; basal transcription; RNA polymerases; and spliceosome. In relation to energy metabolism, they found glycolysis; TCA cycle; oxidative metabolism and oxidative phosphorylation; impaired cell-cell interaction, such as extracellular matrix receptor interaction; tight junction; or cell adhesion molecules [49].

Frontal cortex and white matter shared terms, such as ribosome (upregulated genes); GPI-anchored biosynthesis; and ubiquitin-mediated proteolysis (downregulated genes); as well as leukocyte trans-endothelial migration and cell adhesion molecules (downregulated proteins) [49].

A transcriptomic study performed on skin biopsies (four cases paired with three healthy siblings) using microarray technology showed that the *E2F4* gene was overexpressed in CADASIL patients [50]. Although this difference was not significant after correcting for multiple comparisons, the result was replicated in an independent sample of 10 new cases and 8 new controls by qRT-PCR.

*E2F4* is expressed in endothelial cells and VSMCs as proved by the in situ hybridization performed [50]. This protein is associated with endothelial cell migration and involved in the process of intimal hyperplasia (IH) [51], which is the proliferation of VSMCs in the tunica media and their migration into the tunica intima of the vessel. *E2F4* is also part of a complex containing Smad3, which acts as a transducer of TGFβ signals [50].

When performing an analysis of GO terms, a raw *p*-value under 0.01 was considered enriched since none of the analyses reached a significant *p*-value after correction for multiple comparisons. It is interesting to note that there is an observed growth in the network related to vascular development, catabolic and autophagy processes, vesicular machinery, and cell adhesion terms [50].

### 4.3. Microbiome Studies and Progress in the Etiopathogenesis of CADASIL

There is a single study that focuses on the analysis of the microbiome in CADASIL patients, where the gut microbiome of 15 Japanese CADASIL patients and 16 controls was evaluated [52]. CADASIL patients were divided according to whether they had suffered a symptomatic ischemic stroke (*n* = 7) or not (*n* = 8). There was no significant difference in vascular risk factors or nutritional items between CADASIL patients and the controls. The only significant difference was the daily intake of arachidonic acid between CADASIL subgroups, as patients with stroke had a lower intake (*p* = 0.03) [52].

A total of 790 operational taxonomic units (OTUs) was detected after the removal of singletons. No significant difference was observed in α-diversity or β-diversity between CADASIL patients and the controls or between the patients with and without stroke [52].

In CADASIL patients compared with controls, there was a significant increase in abundance of the following: *Lachnospira, Odoribacter, Parvimonas*, unclassified genera belonging to Barnesiellaceae and Lachnospiraceae, unclassified genus belonging to order SHA-98; and a significant decrease in: *Megasphaera* and *Acidaminococcus*. Regarding the OTU level, CADASIL patients showed a significant increase in abundance of 24 OTUs and a significant decrease in 4 [52]. Between CADASIL subgroups, those with a stroke had a significant decrease in abundance of *Phascolarctobacterium* and *Paraprevotella*, a significant increase in abundance of 13 OTUs, and significant decrease in 3 OTUs [52].

The authors highlight some of these genera, such as *Parvimonas*, as they have been associated with ischemic stroke, or their abundance has been correlated with the amount of C-reactive protein in atherosclerotic patients. Moreover, Lachnospiraceae have been reported to stimulate IL-10 and TGFβ production [52].

**Figure 4:**
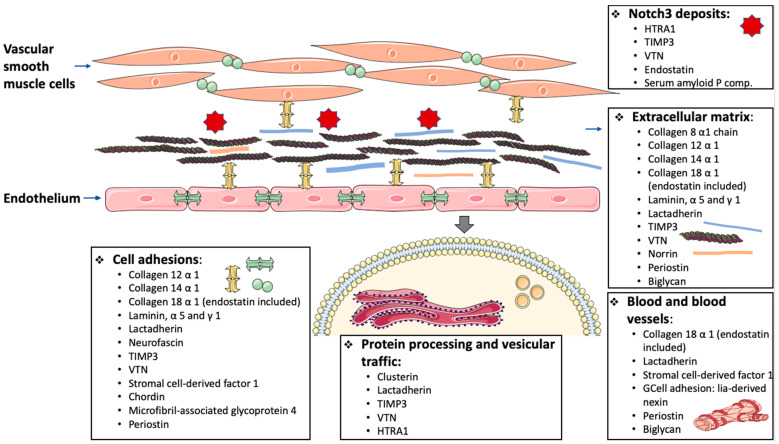
Schematic diagram of those molecules found in the -omics studies regarding its possible aetiopathogenic mechanism. Molecules found in at least two studies and with the same direction of effect have been considered. Graphic created through the smart.servier.com website.

Figure 4 shows a schematic summary of the molecules found in the -omics studies. Molecules found in at least two studies and with the same direction of effect have been considered. 

## 5. Genome-Wide Studies and Progress in Prognostic Assessment

As previously mentioned, we do not have the tools for predicting the prognosis of CADASIL patients, as even monozygotic twins can progress differently [8,9]. 

Different markers have been found to be associated with the deterioration or survival of patients with CADASIL. Dementia and gait disturbance predict a higher degree of disability, whereas the number of lacunar and brain atrophies predict the clinical deterioration of patients with CADASIL [53], which, together with levels of neurofilament light chain, predict progression and survival [54].

A GWAS study conducted in 466 CADASIL patients to find an association with WMH volume did not find any GWAS-significant polymorphisms (SNP) [55]. Nevertheless, a polygenic risk score comprising all SNPs with a *p*-value < 0.5 in the derivation sample was associated with WMH volume in the validation sample after correction for age, sex, and hypertension [55]. 

With these omics techniques, a high prevalence of carriers of pathogenic variants in *NOTCH3* was found within cohorts that do not look at a specific phenotype [17,18,19]. 

This discovery was extremely important, as it showed that the mutational profile in these repositories was different from that of patients diagnosed with CADASIL. 

In 2016, Rutten et al. compared the mutations found in the exome aggregation consortium (ExAC) database and its update, the gnomAD, with the Dutch CADASIL registry (DCR) mutations (comprising 383 individuals with 45 different mutations from 163 families) [17]. They found that the distribution of Dutch CADASIL-causing *NOTCH3* mutations overlapped with those reported worldwide, with most of these mutations affecting exon 4 (Figure 2) [17]. Meanwhile, in ExAC, mutations in exon 4 were three times lower, and mutations in exon 22 were 10 times higher than in the DCR [17]. 

**Figure 5:**
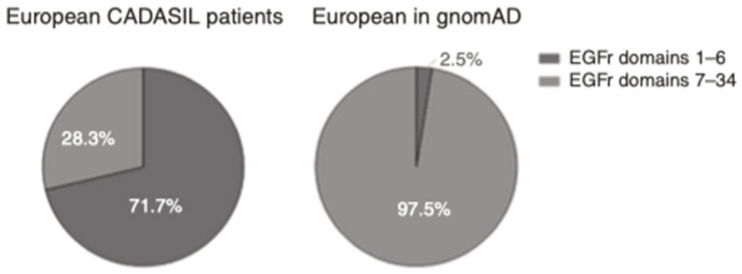
Location of *NOTCH3* mutations in European CADASIL patients versus gnomAD database individuals. Figure extracted from Rutten et al. (2018) [18].

The authors found that mutations in ExAC were mostly clustered in EGFr domains 14–16 and 29–31, whereas reported CADASIL mutations clustered in EGFr domains 1–6 [17]. The study in the gnomAD showed that 2.5% of the *NOTCH3* mutations were in EGFr domains 1–6, whereas in European CADASIL patients, this percentage rose to 71.1% (Figure 5). Studying data from the UKB, 97% of the pathogenic *NOTCH3* variants were found in EGFr domains 7–34 [19].

This led to the hypothesis that mutations outside EGFr domains 1–6, the classical CADASIL mutation location, predispose to a less severe phenotype [17].

Comparing patients with a mutation in EGFr domains 1–6 with patients with a mutation in EGFr domains 7–34 in the DCR, the former had an earlier diagnosis of the disease, a significantly higher WMH load, and a similar trend in the number of subcortical infarcts, and a first stroke at 55 years of age, with a mean survival time of 69 years (as opposed to patients with mutations in EGFr domains 7–34 who have their first stroke at the age of 67 and a mean survival time of 77 years) [17,18].

Comparing individuals with *NOTCH3* pathogenic variants with controls in the UKB [19,20] and the Geisinger DiscovEHR [21], the authors found that subjects with pathogenic variants were correlated with spatial distribution (the anterior temporal lobe and external capsule) and extent of WMH, a higher frequency and number of lacunes and microbleeds, family history of stroke, and vascular dementia.

## 6. Therapeutic Possibilities

Due to the omics research carried out on CADASIL, several molecules with altered levels/expression have emerged, which could therefore be susceptible to pharmacological repositioning. Due to their relevance in the different studies, different authors have highlighted several molecules and studied them in-depth: Notch3, VTN, TIMP3, and Serum amyloid P-component. We have studied the possible treatments for pharmacological repositioning through DrugBank. For Notch3, endostatin, HTRA1, and E2F4 we have not found any available drugs. See Table 2.

## 7. Limitations

The major limitation is that most of the studies were conducted on a very small number of patients, which limits the possibility of achieving statistically significant results adjusted for multiple comparisons. This may be partly due to the low frequency of the disease and its target tissue. 

To overcome this low sample size, some authors performed studies directly in brain vessels, using sex- and age-matched subjects, or performed a similar analysis in animal models to support the findings observed. In fact, most of the molecules that overlapped in the different studies follow the same direction, supporting the findings. Moreover, in most studies, the molecules involved have common functions/pathways.

For the microbiome study, the sample size was small, and there was no replication study. The authors argue that even with small samples, other studies have reported robust evaluation of gut dysbiosis. The findings should nonetheless be taken with caution.

A limitation of the review processes used is that studies using omics other than the ones contained in the keywords may have been excluded. However, the studies with the highest volume of research for understanding CADASIL are in the field of genomics, transcriptomics, and proteomics. 

## 8. Discussion and Conclusions

Due to the introduction of omics techniques in CADASIL studies, we have been able to gain insight into several aspects of the disease.

Nowadays, the diagnosis of CADASIL is more efficient, leading to a lower cost and time for analyzing more exons, which means that a larger number of patients can be screened in a more comprehensive way.

From an epidemiological point of view, we have gone from establishing a minimum CADASIL prevalence of 1.32–4.1/100,000 adults [14,15,16] to a prevalence of 1.4–3.4/1000 carriers of pathological variants in *NOTCH3* [17,18,19,20,21] and 9/1000 in Taiwan [22].

Currently, comparing patients with a mutation in the EGFr domains 1–6 with patients with a mutation in EGFr domains 7–34 in the above-mentioned biobanks, subjects with pathogenic variants were correlated with spatial distribution (the anterior temporal lobe and external capsule) and extent of WMH, a higher frequency and number of lacunes and microbleeds, family history of stroke, and vascular dementia [19,20,21].

This finding highlights the importance of the use of these technologies. The role of mutations considered pathogenic has been questioned. If they all produce CADASIL, then CADASIL is no longer a rare disease. Therefore, depending on the altered structural or functional domain, the patient could have a more or less torpid course. Another hypothesis is that perhaps not all pathogenic variants cause CADASIL. Since SNPs in the *NOTCH3* gene were not found to be associated with lacunar stroke or WMH volume [56], maybe only the variants that affect the number of cysteines in EGFr are risk factors for developing small vessel disease, instead of CADASIL. 

From the etiology perspective, omics techniques have made it possible to find molecules whose levels/expression are altered in patients with CADASIL, and which may be amenable to pharmacological repositioning. The most highlighted molecules by the authors in the articles were Notch3, HTRA1, TIMP3, VTN, endostatin, and serum amyloid P-component. The different studies showed that these proteins are co-localized with Notch3, supporting the etiopathogenic interest in them.

Mutations in HTRA1 are also the cause of small vessel cerebral arteriopathy in heterozygosis and homozygosis. In the latter case, they cause CARASIL (Cerebral Autosomal Recessive Arteriopathy with Subcortical Infarcts and Leukoencephalopathy), leading to TGFβ signaling impairment. TGFβ is closely related to small vessel disease in the brain [57]. The fact that several of the substrates of this enzyme are elevated in patients with CADASIL compared with controls, as in the case of VTN, suggests that its proteolytic activity may be diminished [46], as is the case of CARASIL [58]. 

The remaining molecules found in the proteomic studies, with the same direction of effect in at least two different studies, are likely to be of special interest in the disease. In fact, they have been related to angiogenesis, protein processing and vesicular trafficking, nervous system, or extracellular matrix, among others. To confirm their interest, targeted studies on these proteins would be of important value. Those proteins with contradictory effects in the studies reviewed may be false-positive associations and, therefore, should be taken with caution. 

From transcriptomic studies, *E2F4* has been found to be overexpressed in CADASIL patients, not knowing whether this elevated expression is due to a compensatory mechanism for lack of protein production or elevated levels of the protein. 

E2F4 belongs to the E2F family of transcription factors. E2F members have been involved in neuronal migration, and activation of E2Fs in VSMCs promotes migration [59]. E2F4 is involved in the process of IH [51]. Mice lacking E2F4 exhibit increased IH following arterial damage [51].

Besides, a diminished expression of *E2F4* attenuates the endothelial cell migration, and its subsequent overexpression could rescue normal endothelial migration [59].

Likewise, E2F4 is part of the signaling pathway of TGFβ [60]. As we have just mentioned, TGFβ is related to cerebral small vessel disease. On the one hand, mutations in *HTRA1* produce a TGFβ signaling impairment and lead to the appearance of CARASIL. On the other hand, in CADASIL, HTRA1, and LTBP-1 (protein that regulates bioavailability of TGFβ [57]) are present in GOMs, the histopathological hallmark of the disease.

It is, therefore, important that all this information help us understand the reason for the alterations found in CADASIL, such as changes in cell adhesions and components of the extracellular matrix or angiogenesis, as well as highlighting other pathways that thus far only a few studies have considered, and which can be decisive, such as alterations in autophagy, mitochondria, or the TGFβ signaling pathway. This could encourage researchers to find drugs that could directly modify pathways that are clearly involved in CADASIL, instead of looking for drugs that target a specific molecule.

Therefore, thanks to this vanguard technology, progress has been made in understanding the extent of the disease and its etiopathogenesis, but there is still a long way to go before we can fully comprehend what happens in CADASIL. Omic studies will allow further investigation of the etiopathogenesis of the disease in a cost-effective manner, since the costs of this type of technology are becoming increasingly lower, allowing the analysis of a larger number of patients and molecules at once, which with conventional techniques would entail a high expenditure of resources and time. In addition, it will allow the exploration of new working hypotheses in an unbiased manner and develop disease-modifying drugs in the future. 

## Figures and Tables

**Table 1:** Proteomic analysis of human brain vessels. Proteins whose levels were different between patients with CADASIL and controls in the study by Arboleda-Velasquez et al. [43], Monet-Leprêtre et al. [44], and Nagatoshi et al. [47].

Protein Name	Change AV	Change ML	Change N	Molecular Functions and Biological Process
**Annexin A2 pseudogene 2 [43]**	+			
**Clusterin [43]**	+	+		**Protein processing and vesicular traffic**: response to misfolded protein, chaperone-mediated protein folding, chaperone-mediated protein complex assembly, ubiquitin protein ligase binding, negative regulation of response to endoplasmic reticulum stress **Nervous system**: regulation of neuron death, microglial cell proliferation and activation, central nervous system myelin maintenance **Proteins related to Alzheimer’s disease**: amyloid-beta binding, regulation of amyloid-beta clearance and negative regulation of amyloid-beta formation; tau protein binding, positive regulation of tau-protein kinase activity, positive regulation of neurofibrillary tangle assembly **Survival**: negative regulation of intrinsic apoptotic signaling pathway in response to DNA damage
**Collagen 12 α 1 [43]**	+	+	+	**Extracellular matrix**: extracellular matrix structural constituent conferring tensile strength **Cell adhesion**
**Collagen 14 α 1 [43]**	+	+		**Extracellular matrix**: extracellular matrix structural constituent conferring tensile strength **Cell adhesion**
**Collagen 18 α 1 (endostatin included) [43]**	+	+		**Extracellular matrix**: extracellular matrix structural constituent conferring tensile strength, extracellular matrix organization **Cell adhesion** **Blood and blood vessels**: angiogenesis, endothelial cell morphogenesis, response to hydrostatic pressure
**Collagen 1 α 2 [43]**	+			**Protein processing and vesicular traffic**: rho protein signal transduction **Extracellular matrix**: collagen fibril organization, extracellular matrix assembly **Blood and blood vessel**: blood vessel development, regulation of blood pressure, platelet-derived growth factor binding **Smooth muscle**: rho protein signal transduction **TGFβ signaling**: TGFβ receptor signaling pathway, SMAD binding
**Hemoglobin, α [43]**	-	-		**Survival**: positive regulation of cell death, oxygen transport
**Perlecan [43]**	+			**Protein processing and vesicular traffic**: receptor-mediated endocytosis **Extracellular matrix**: extracellular matrix structural constituent conferring compression resistance **Blood and blood vessel**: angiogenesis **General development**: animal organ morphogenesis, tissue development
**Internexin, α [43]**	-	-		**Cytoskeleton**: neurofilament and intermediate filament cytoskeleton organization, postsynaptic actin cytoskeleton organization **Nervous system**: nervous system development, structural constituent of postsynaptic actin **General development**: cell differentiation, multicellular organism development
**Laminin, α 5 [43]**	+	+		**Extracellular matrix**: extracellular matrix structural constituent **Cell adhesion**: integrin binding, integrin-mediated signaling pathway, cell adhesion **Nervous system**: neural crest cell migration, axon guidance **General development**: animal organ morphogenesis, tissue development, regulation of cell migration
**Laminin, γ 1 [43]**	+	+		**Extracellular matrix**: extracellular matrix structural constituent and disassembly **Cell adhesion**: hemidesmosome assembly, cell adhesion **Nervous system**: maintenance of blood–brain barrier **General development**: animal organ morphogenesis, tissue development, cell migration
**Lactadherin (medin included) [43]**	+	+	+	**Protein processing and vesicular traffic**: phagocytosis recognition, engulfment, and positive regulation **Extracellular matrix**: Extracellular matrix structural constituent **Cell adhesion**: integrin binding, cell adhesion **Blood and blood vessel**: angiogenesis
**Smooth muscle myosin heavy chain [43]**	+			**Cytoskeleton**: actin binding **Mucle contraction**: calmodulin binding Others: elastic fiber assembly, structural constituent of muscle
**Neurofilament protein, light polypeptide [43]**	-	-		**Cytoskeleton**: microtubule cytoskeleton organization, neurofilament bundle assembly, postsynaptic intermediate filament cytoskeleton organization, neurofilament cytoskeleton organization **Nervous system**: anterograde and retrograde axonal transport, axonal transport of mitochondrion, hippocampus and cerebral cortex development, negative regulation of neuron apoptotic process, neuron projection morphogenesis, positive regulation of axonogenesis, axon development
**Neurofilament 3 [43]**	-			**Cytoskeleton**: neurofilament bundle assembly
**Neurofascin [43]**	-	-		**Cell adhesion** **Nervous system**: transmission of nerve impulse, axon guidance, myelination, synapse organization
**Leucine rich repeat proteoglycans [43]**	+			**Extracellular matrix**: Extracellular matrix structural constituent **Survival**: Cell aging
**Solute carrier family 4**	-			**Protein processing and vesicular traffic**: transmembrane transport **Cytoskeleton**: ankyrin binding **Blood and blood vessel**: blood coagulation, hemoglobin binding
**Vinculin [43]**	+			**Protein processing and vesicular traffic**: ubiquitin protein ligase binding **Cytoskeleton**: actin filament binding Cell adhesion: adherens junction assembly, apical junction assembly, regulation of focal adhesion assembly, epithelial cell-cell adhesion, regulation of protein localization to adherens junction, beta-catenin binding, alpha-catenin binding, cadherin binding **Nervous system**: axon extension **General development**: regulation of cell migration
**Serum amyloid P-component [44]**	+	+	+	Others: negative regulation of glycoprotein metabolic process, low-density lipoprotein particle binding
**TIMP3 [44]**	+	+		**Protein processing and vesicular traffic**: negative regulation of membrane protein ectodomain proteolysis **Cell adhesion**: negative regulation of ERK1 and ERK2 cascade Others: metalloendopeptidase inhibitor activity
**VTN [44]**	+	+	+	**Protein processing and vesicular traffic**: endocytosis, scavenger receptor activity **Extracellular matrix**: extracellular matrix structural constituent **Cell adhesion**: cell-matrix adhesion, positive regulation of smooth muscle cell migration, cell adhesion, integrin-binding **General development**: cell migration
**Stromal cell-derived factor 1 [44]**	+	+		**Cell adhesion**: positive regulation of cell adhesion, integrin-binding **Blood and blood vessel**: positive regulation of endothelial cell proliferation **Nervous system**: neuron migration, brain development, positive regulation of neuron differentiation, positive regulation of axon extension involved in axon guidance **Survival**: response to hypoxia, negative regulation of intrinsic apoptotic signaling pathway in response to DNA damage
**Chordin [44]**	+	+		**Cell adhesion**: positive regulation of cell adhesion **General development**: negative regulation of cell migration
**Norrin [44]**	+	+		**Extracellular matrix**: extracellular matrix-cell signaling
**Serine protease 23 [44]**	+	+		**Protein processing and vesicular traffic**: proteolysis
**High-temperature requirement protein A1 [44]**	+	+		**Protein processing and vesicular traffic**: serine-type endopeptidase activity **TGFβ signaling**: negative regulation of TGFβ receptor signaling pathway
**GCell adhesion: lia-derived nexin [44]**	+	+		**Protein processing and vesicular traffic**: serine-type endopeptidase inhibitor activity, secretory granule organization, negative regulation of protein processing **Blood and blood vessel**: negative regulation of blood coagulation **Nervous system**: nervous system development, negative regulation of cell population proliferation, glutamatergic regulation of synaptic transmission, positive regulation of astrocyte differentiation **General development**: cell differentiation
**Cell migration-inducing and hyaluronan-binding protein [44]**		+		**General development**: positive regulation of cell migration Others: clathrin heavy chain binding
**Olfactomedin-like 3 [44]**	+	+		**General development**: multicellular organism development
**Collagen VIII α1 chain [44]**		+		**Extracellular matrix**: extracellular matrix structural constituent structural constituent **Cell adhesion**: cell adhesion **Blood and blood vessel**: angiogenesis
**Microfibril-associated glycoprotein 4 [44]**		+		**Extracellular matrix**: extracellular matrix structural constituent, regulation of collagen metabolic process, elastic fiber assembly **Cell adhesion**: cell adhesion
***NOTCH3* [44]**	+	+	+	**Cell adhesion**: cadherin binding **Nervous system**: axon guidance **General development**: multicellular organism development, cell differentiation
**Annexin A2 [47]**			+	**Protein processing and vesicular traffic**: positive regulation of protein phosphorylation, negative regulation of endopeptidase activity, negative regulation of catalytic activity, vesicle budding from the membrane, positive regulation of vacuole organization, positive regulation of vesicle fusion, positive regulation of receptor recycling **Blood and blood vessel**: angiogenesis **Extracellular matrix**: collagen fibril organization **Cell adhesion**: cell-cell adhesion
**Periostin [47]**		+	+	**Survival**: response to hypoxia **Cell adhesion**: negative regulation of cell-matrix adhesion; cell adhesion **Blood and blood vessel**: regulation of systemic arterial blood pressure **Notch signaling**: regulation of Notch signaling pathway **General development**: Tissue development **Smooth muscle**: positive regulation of smooth muscle cell migration **Extracellular matrix**: extracellular matrix organization, extracellular matrix structural constituent **TGFβ signaling**: cellular response to transforming growth factor-beta stimulus **Nervous system**: neuron projection extension
**Complement C3 [47]**			+	**Protein processing and vesicular traffic**: negative regulation of endopeptidase activity, positive regulation of receptor-mediated endocytosis, positive regulation of phagocytosis, engulfment, positive regulation of apoptotic cell clearance **Blood and blood vessel**: positive regulation of vascular endothelial growth factor production **Proteins related to Alzheimer’s disease**: amyloid-beta clearance **Nervous system**: neuron remodeling
**Plectin [47]**		-	+	**Cytoskeleton**: Intermediate filament cytoskeleton organization, actin-binding, ankyrin bindin **Cell adhesion**: hemidesmosome assembly, cadherin binding
**Biglycan [47]**		+	+	**Extracellular matrix**: extracellular matrix structural constituent **Blood and blood vessel**: blood vessel remodeling
**Gelsolin [47]**		-	+	**Protein processing and vesicular traffic**: positive regulation of cysteine-type endopeptidase activity involved in apoptotic signaling pathway, regulation of receptor clusterin, positive regulation of protein processing in the phagocytic vesicle, vesicle-mediated transport **Cytoskeleton**: actin filament reorganization, actin-binding **Cell adhesion**: regulation of cell adhesion **Nervous system**: oligodendrocyte development **General development**: cell projection organization Others: response to muscle stretch

**Table 2:** Potential drugs for the targeted molecules found in omic studies. For Notch3, endostatin, HTRA1, E2F4, we have not found any available drugs.

Target	Drug	Description
**VTN**	Abciximab	Fab fragment of the chimeric human-murine monoclonal antibody 7E3. Abciximab binds to the glycoprotein (GP) IIb/IIIa receptor of human platelets and inhibits platelet aggregation by preventing the binding of fibrinogen, von Willebrand factor, and other adhesive molecules. It also binds to the vitronectin (αvβ3) receptor found on platelets and vessel wall endothelial and smooth muscle cells
	Others: copper, zinc, zinc acetate, chloride	
**TIMP3**	Pimagedine	Pimagedine has been developed by Synvista Therapeutics, Inc for the treatment of diabetic kidney disease. It is an advanced glycation end-product inhibitor that manages diabetic nephropathy, either alone or in combination with other therapies. It is beneficial in treating patients with diabetic nephropathy.
**Serum amyloid P-component**	Methyl 4,6-O-[(1R)-1-carboxyethylidene]-beta-D-galactopyranoside, Bis-1,2-{[(Z)-2-carboxy-2-methyl-1,3-dioxane]-5-yloxycarbamoyl}-ethane, copper, zinc, zinc acetate, chloride	

## References

[1] Coto E., Menéndez M., Navarro R., García-Castro M., Alvarez V. (2006). A new de novo Notch3 mutation causing CADASIL. Eur. J. Neurol..

[2] Opherk C., Duering M., Peters N., Karpinska A., Rosner S., Schneider E., Bader B., Giese A., Dichgans M. (2009). CADASIL mutations enhance spontaneous multimerization of NOTCH3. Hum. Mol. Genet..

[3] Duering M., Karpinska A., Rosner S., Hopfner F., Zechmeister M., Peters N., Kremmer E., Haffner C., Giese A., Dichgans M. (2011). Co-aggregate formation of CADASIL-mutant NOTCH3: A single-particle analysis. Hum. Mol. Genet..

[4] Chabriat H., Vahedi K., Iba-Zizen M.T., Joutel A., Nibbio A., Nagy T.G., Krebs M.O., Julien J., Dubois B., Ducrocq X. (1995). Clinical spectrum of CADASIL: A study of 7 families. Cerebral autosomal dominant arteriopathy with subcortical infarcts and leukoencephalopathy. Lancet.

[5] Markus H.S., Martin R.J., Simpson M.A., Dong Y.B., Ali N., Crosby A.H., Powell J.F. (2002). Diagnostic strategies in CADASIL. Neurology.

[6] O’Sullivan M., Jarosz J.M., Martin R.J., Deasy N., Powell J.F., Markus H.S. (2001). MRI hyperintensities of the temporal lobe and external capsule in patients with CADASIL. Neurology.

[7] Rumbaugh J.A., LaDuca J.R., Shan Y., Miller C.A. (2000). CADASIL: The dermatologic diagnosis of a neurologic disease. J. Am. Acad. Dermatol..

[8] Mykkänen K., Junna M., Amberla K., Bronge L., Kääriäinen H., Pöyhönen M., Kalimo H., Viitanen M. (2009). Different clinical phenotypes in monozygotic CADASIL twins with a novel Notch3 mutation. Stroke.

[9] Ceroni M., Poloni T.E., Tonietti S., Fabozzi D., Uggetti C., Frediani F., Simonetti F., Malaspina A., Alimonti D., Celano M. (2000). Migraine with aura and white matter abnormalities: Notch3 mutation. Neurology.

[10] Horgan R.P., Kenny L.C. (2011). ‘Omic’ technologies: Proteomics and metabolomics learning objectives: Ethical issues. Obstet. Gynaecol..

[11] Orset C., De Grange P., Rousselet E., Ramsay L., Quill M. (2019). Blood transcriptomic biomarker as a surrogate of ischemic brain gene expression. Ann. Clin. Transl. Neurol..

[12] Chong M., Sjaarda J., Pigeyre M., Mohammadi-Shemirani P., Lali R., Shoamanesh A., Gerstein H.C., Paré G. (2019). Novel drug targets for ischemic stroke identified through Mendelian randomization analysis of the blood proteome. Circulation.

[13] Razvi S.S.M.M., Davidson R., Bone I., Muir K.W. (2005). The prevalence of cerebral autosomal dominant arteriopathy with subcortical infarcts and leucoencephalopathy (CADASIL) in the west of Scotland. J. Neurol. Neurosurg. Psychiatry.

[14] Narayan S.K., Kalaria R.N. (2012). The minimum prevalence of CADASIL in northeast England. Neurology.

[15] Moreton F.C., Razvi S.S.M.M., Davidson R., Muir K.W. (2014). Changing clinical patterns and increasing prevalence in CADASIL. Acta Neurol. Scand..

[16] Bianchi S., Zicari E., Carluccio A., Di Donato I., Pescini F., Nannucci S., Valenti R., Ragno M., Inzitari D., Pantoni L. (2015). CADASIL in central Italy: A retrospective clinical and genetic study in 229 patients. J. Neurol..

[17] Rutten J.W., Dauwerse H.G., Gravesteijn G., van Belzen M.J., van der Grond J., Polke J.M., Bernal-Quiros M., Lesnik Oberstein S.A.J., Van Belzen M.J., Der Grond V. (2016). Archetypal NOTCH3 mutations frequent in public exome: Implications for CADASIL. Ann. Clin. Transl. Neurol..

[18] Rutten J.W., Van Eijsden B.J., Duering M., Jouvent E., Opherk C., Pantoni L., Federico A., Dichgans M., Markus H.S., Chabriat H. (2018). The effect of NOTCH3 pathogenic variant position on CADASIL disease severity: NOTCH3 EGFr 1–6 pathogenic variant are associated with a more severe phenotype and lower survival compared with EGFr 7–34 pathogenic variant. Genet. Med..

[19] Rutten J.W., Hack R.J., Duering M., Gravesteijn G., Dauwerse J., Overzier M., van den Akker E.B., Slagboom E., Holstege H., Nho K. (2020). Broad phenotype of cysteine altering NOTCH3 variants in UK Biobank: CADASIL to nonpenetrance. Neurology.

[20] Cho B.P.H., Nannoni S., Harshfield E.L., Tozer D., Gräf S., Bell S., Markus H.S. (2021). NOTCH3 variants are more common than expected in the general population and associated with stroke and vascular dementia: An analysis of 200,000 participants. J. Neurol. Neurosurg. Psychiatry.

[21] Hack R.J., Rutten J.W., Person T.N., Li J., Khan A. (2020). Cysteine-altering NOTCH3 variants are a risk factor for stroke in the elderly population. Stroke.

[22] Lee Y., Chung C., Chang M., Wang S. (2020). NOTCH3 cysteine-altering variant is an important risk factor for stroke in the Taiwanese population. Neurology.

[23] Meng H., Zhang X., Yu G., Lee S.J., Chen Y.E., Prudovsky I., Wang M.M. (2012). Biochemical characterization and cellular effects of CADASIL mutants of NOTCH3. PLoS ONE.

[24] Joutel A. (2011). Pathogenesis of CADASIL: Transgenic and knock-out mice to probe function and dysfunction of the mutated gene, Notch3, in the cerebrovasculature. BioEssays.

[25] Joutel A., Andreux F., Gaulis S., Domenga V., Cecillon M., Battail N., Piga N., Chapon F., Godfrain C., Tournier-Lasserve E. (2000). The ectodomain of the Notch3 receptor accumulates within the cerebrovasculature of CADASIL patients. J. Clin. Investig..

[26] Ishiko A., Shimizu A., Nagata E., Takahashi K., Tabira T., Suzuki N. (2006). Notch3 ectodomain is a major component of granular osmiophilic material (GOM) in CADASIL. Acta Neuropathol..

[27] Ueda A., Hirano T., Takahashi K., Kurisaki R., Hino H., Uyama E., Uchino M. (2009). Detection of granular osmiophilic material of cerebral autosomal dominant arteriopathy with subcortical infarcts and leukoencephalopathy by light microscopy in frozen sections: Scientific correspondence. Neuropathol. Appl. Neurobiol..

[28] Ruchoux M.M., Maurage C.A. (1998). Endothelial changes in muscle and skin biopsies in patients with CADASIL. Neuropathol. Appl. Neurobiol..

[29] Lewandowska E., Leszczyńska A., Wierzba-Bobrowicz T., Skowrońska M., Mierzewska H., Pasennik E., Członkowska A. (2006). Ultrastructural picture of blood vessels in muscle and skin biopsy in CADASIL. Folia Neuropathol..

[30] Ghosh M., Balbi M., Hellal F., Dichgans M., Lindauer U., Plesnila N. (2015). Pericytes are involved in the pathogenesis of cerebral autosomal dominant arteriopathy with subcortical infarcts and leukoencephalopathy. Ann. Neurol..

[31] Okeda R., Arima K., Kawai M. (2002). Arterial changes in cerebral autosomal dominant arteriopathy with subcortical infarcts and leukoencephalopathy (CADASIL) in relation to pathogenesis of diffuse myelin loss of cerebral white matter: Examination of cerebral medullary arteries by reconstruction of serial sections of an autopsy case. Stroke.

[32] Brulin P., Godfraind C., Leteurtre E., Ruchoux M.M. (2002). Morphometric analysis of ultrastructural vascular changes in CADASIL: Analysis of 50 skin biopsy specimens and pathogenic implications. Acta Neuropathol..

[33] Ruchoux M.M., Chabriat H., Baudrimont M., Tournier-Lasserve E. (1994). Presence of ultrastructural arterial lesions in muscle and skin vessels of patients with CADASIL. Stroke.

[34] Tikka S., Peng Ng Y., Di Maio G., Mykkänen K., Siitonen M., Lepikhova T., Pöyhönen M., Viitanen M., Virtanen I., Kalimo H. (2012). CADASIL mutations and ShRNA silencing of NOTCH3 affect actin organization in cultured vascular smooth muscle cells. J. Cereb. Blood Flow Metab..

[35] Ruchoux M.M., Domenga V., Brulin P., Maciazek J., Limol S., Tournier-Lasserve E., Joutel A. (2003). Transgenic mice expressing mutant Notch3 develop vascular alterations characteristic of cerebral autosomal dominant arteriopathy with subcortical infarcts and leukoencephalopathy. Am. J. Pathol..

[36] Haritunians T., Chow T., De Lange R.P.J.J., Nichols J.T., Ghavimi D., Dorrani N., St Clair D.M., Weinmaster G., Schanen C., St. Clair D.M. (2005). Functional analysis of a recurrent missense mutation in Notch3 in CADASIL. J. Neurol. Neurosurg. Psychiatry.

[37] Baron-Menguy C., Domenga-Denier V., Ghezali L., Faraci F.M., Joutel A. (2017). Increased Notch3 activity mediates pathological changes in structure of cerebral arteries. Hypertension.

[38] Hanemaaijer E.S., Panahi M., Swaddiwudhipong N., Tikka S., Winblad B., Viitanen M., Piras A., Behbahani H. (2018). Autophagy-lysosomal defect in human CADASIL vascular smooth muscle cells. Eur. J. Cell Biol..

[39] Lewandowska E., Felczak P., Buczek J., Gramza K., Rafałowska J. (2014). Blood vessel ultrastructural picture in a CADASIL patient diagnosed at an advanced age. Folia Neuropathol..

[40] Viitanen M., Sundström E., Baumann M., Poyhonen M., Tikka S., Behbahani H. (2013). Experimental studies of mitochondrial function in CADASIL vascular smooth muscle cells. Exp. Cell Res..

[41] Lewandowska E., Wierzba-Bobrowicz T., Buczek J., Gromadzka G., Dziewulska D. (2013). CADASIL patient with extracellular calcium deposits. Folia Neuropathol..

[42] De La Peña P., Bornstein B., Del Hoyo P., Fernández-Moreno M.A., Martín M.A., Campos Y., Gómez-Escalonilla C., Molina J.A., Cabello A., Arenas J. (2001). Mitochondrial dysfunction associated with a mutation in the Notch3 gene in a CADASIL family. Neurology.

[43] Arboleda-Velasquez J.F., Manent J., Hyun J., Tikka S., Ospina C., Vanderburg C.R., Lee J.H., Tikka S., Ospina C., Vanderburg C.R. (2011). Hypomorphic Notch 3 alleles link Notch signaling to ischemic cerebral small-vessel disease. Proc. Natl. Acad. Sci. USA.

[44] Monet-Leprêtre M., Haddad I., Baron-Menguy C., Fouillot-Panchal M., Riani M., Domenga-Denier V., Dussaule C., Cognat E., Vinh J., Joutel A. (2013). Abnormal recruitment of extracellular matrix proteins by excess Notch3 ECD: A new pathomechanism in CADASIL. Brain.

[45] Capone C., Dabertrand F., Baron-Menguy C., Chalaris A., Ghezali L., Domenga-Denier V., Schmidt S., Huneau C., Rose-John S., Nelson M.T. (2016). Mechanistic insights into a TIMP3-sensitive pathway constitutively engaged in the regulation of cerebral hemodynamics. Elife.

[46] Zellner A., Scharrer E., Arzberger T., Oka C., Domenga-Denier V., Joutel A., Lichtenthaler S.F., Müller S.A., Dichgans M., Haffner C. (2018). CADASIL brain vessels show a HTRA1 loss-of-function profile. Acta Neuropathol..

[47] Nagatoshi A., Ueda M., Ueda A., Tasaki M., Inoue Y., Ma Y., Masuda T., Mizukami M., Matsumoto S., Kosaka T. (2017). Serum amyloid P component: A novel potential player in vessel degeneration in CADASIL. J. Neurol. Sci..

[48] Primo V., Graham M., Bigger-Allen A.A., Chick J.M., Ospina C., Quiroz Y.T., Manent J., Gygi S.P., Lopera F., D’Amore P.A. (2016). Blood biomarkers in a mouse model of CADASIL. Brain Res..

[49] Jenoe P., Bonati L., Engelter S., Lyrer P. (2019). Combined transcriptomic and proteomic analyses of cerebral frontal lobe tissue identified RNA metabolism dysregulation as one potential pathogenic mechanism in cerebral autosomal dominant arteriopathy with subcortical infarcts and leukoencephalopathy (CADASIL). Curr. Neurovasc. Res..

[50] Muiño E., Maisterra O., Balado J.J., Cullell N., Carrera C., Aguila N.P.T., Márquez J.C., Fabrega C.G., Lledós M., Sánchez J.G. (2021). Genome-wide transcriptome study in skin biopsies reveals an association of E2F4 with cadasil and cognitive impairment. Sci. Rep..

[51] Giangrande P.H., Zhang J.X., Tanner A., Eckhart A.D., Rempel R.E., Andrechek E.R., Layzer J.M., Keys J.R., Hagen P.O., Nevins J.R. (2007). Distinct roles of E2F proteins in vascular smooth muscle cell proliferation and intimal hyperplasia. Proc. Natl. Acad. Sci. USA.

[52] Matsuura J., Inoue R., Takagi T., Wada S., Watanabe A., Koizumi T., Mukai M., Mizuta I., Naito Y., Mizuno T. (2019). Analysis of gut microbiota in patients with cerebral autosomal dominant arteriopathy with subcortical infarcts and leukoencephalopathy. J. Clin. Biochem. Nutr..

[53] Chabriat H., Hervé D., Duering M., Godin O., Jouvent E., Opherk C., Alili N., Reyes S., Jabouley A., Zieren N. (2015). Predictors of clinical worsening in cerebral autosomal dominant arteriopathy with subcortical infarcts and leukoencephalopathy: Prospective cohort study. Stroke.

[54] Gravesteijn G., Rutten J.W., Verberk I.M.W., Böhringer S., Liem M.K., van der Grond J., Aartsma-Rus A., Teunissen C.E., Lesnik Oberstein S.A.J. (2019). Serum neurofilament light correlates with CADASIL disease severity and survival. Ann. Clin. Transl. Neurol..

[55] Opherk C., Gonik M., Duering M., Malik R., Jouvent E., Hervé D., Adib-Samii P., Bevan S., Pianese L., Silvestri S. (2014). Genome-wide genotyping demonstrates a polygenic risk score associated with white matter hyperintensity volume in CADASIL. Stroke.

[56] Rutten-Jacobs L.C.A., Traylor M., Adib-Samii P., Thijs V., Sudlow C., Rothwell P.M., Boncoraglio G., Dichgans M., Bevan S., Meschia J. (2015). Common NOTCH3 variants and cerebral small-vessel disease. Stroke.

[57] Müller K., Courtois G., Ursini M.V., Schwaninger M. (2017). New insight into the pathogenesis of cerebral small-vessel diseases. Stroke.

[58] Nozaki H., Kato T., Nihonmatsu M., Saito Y., Mizuta I., Noda T., Koike R., Miyazaki K., Kaito M., Ito S. (2016). Distinct molecular mechanisms of HTRA1 mutants in manifesting heterozygotes with CARASIL. Neurology.

[59] Gu L., Hitzel J., Moll F., Kruse C., Malik R.A., Preussner J., Looso M., Leisegang M.S., Steinhilber D., Brandes R.P. (2016). The histone demethylase PHF8 is essential for endothelial cell migration. PLoS ONE.

[60] Chen C.R., Kang Y., Siegel P.M., Massagué J. (2002). E2F4/5 and P107 as Smad cofactors linking the TGFβ receptor to c-myc repression. Cell.

